# Associations of HIV persistence, cigarette smoking, inflammation, and pulmonary dysfunction in people with HIV on antiretroviral therapy

**DOI:** 10.1097/MD.0000000000029264

**Published:** 2022-07-08

**Authors:** Joshua Cyktor, Shulin Qin, Brittany Staines, Mehdi Nouraie, Meghan Fitzpatrick, Cathy Kessinger, Rebecca DeSensi, Laurence Huang, Charles R. Rinaldo, Lawrence Kingsley, Phyllis C. Tien, John W. Mellors, Alison Morris

**Affiliations:** a Department of Medicine, University of Pittsburgh, PA, USA; b Department of Medicine, University of California San Francisco, CA, USA; c Department of Infectious Diseases and Microbiology, Graduate School of Public Health, University of Pittsburgh, PA, USA; d Department of Veterans Affairs Medical Center, San Francisco, CA, USA; e Department of Immunology, University of Pittsburgh, PA, USA.

**Keywords:** cytokines, HIV-1 reservoirs, inflammation, pulmonary function, single copy assay, smoking

## Abstract

We aimed to investigate the relationship between measures of HIV persistence with antiretroviral therapy (ART) and cigarette smoking, systemic markers of inflammation, and pulmonary function.

Retrospective study of 82 people with HIV (PWH) on ART for a median of 6.9 years (5.6–7.8) and plasma HIV RNA levels <50 copies/mL.

HIV DNA and cell-associated HIV RNA (CA-RNA) were measured in peripheral blood mononuclear cells (PBMC) and plasma HIV RNA was measured by single-copy assay (SCA). Plasma levels of 17 inflammatory mediators were measured by Bio-Plex, and standard pulmonary function tests (PFT) were performed in all participants.

Median age was 52 years and 41% were women. Most had preserved CD4^+^ T cell counts (median (IQR) 580 (361–895) cells/mm^3^). Median plasma HIV RNA was 1.3 (0.7–4.6) copies/mL, and median levels of HIV DNA and CA-RNA in PBMC were 346 (140–541) copies and 19 (3.7–49) copies per 1 million PBMC, respectively. HIV DNA was higher in smokers than in nonsmokers (*R* = 0.3, *P* < 0.05), and smoking pack-years positively correlated with HIV DNA and CA-RNA (*R* = 0.3, *P* < 0.05 and *R* = 0.4, *P* < 0.01, respectively). HIV DNA, CA-RNA, and plasma HIV RNA were not significantly associated with any measure of pulmonary function or inflammation.

Cigarette smoking was associated with HIV DNA and CA-RNA levels in blood, but measures of HIV persistence were not associated with pulmonary function or inflammation.

## 1. Introduction

With the success of modern antiretroviral therapy (ART), people with HIV are living longer, healthier lives and developing nonAIDS comorbidities at an earlier age than those without HIV infection. Long-term inflammation from immune dysfunction and factors such as smoking may be important contributors.^[1]^ More PWH develop chronic obstructive pulmonary disease (COPD) and lung cancer^[2,3]^ than those without HIV. Rates of cigarette smoking in PWH are about double that of the general US population,^[4]^ and long-term exposure to cigarette smoke can result in chronic oxidative stress, immunosuppression, and a persistent, systemic inflammatory response that is enhanced by HIV.^[5]^

Chronic inflammation is a hallmark of HIV infection and is strongly associated with pulmonary dysfunction.^[6,7]^ This inflammation may be due to persistent antigen stimulation, microbial translocation by disruption of the gut mucosa, co-infections, and/or cumulative ART toxicity^[8,9]^; HIV infection is associated with lymphocytic alveolitis,^[10]^ and HIV gp120 can cause airway damage,^[11]^ suggesting that HIV persistence could directly stimulate pulmonary inflammation. Indeed, plasma HIV RNA levels >200,000 copies/mL are associated with obstructive lung disease.^[12]^ ART can reduce some circulating inflammatory mediators (IL-2, IL-5, IL-7, IL-9, IL12p70, TNF) to near normal levels although inflammation can persist and the degree of chronic inflammation is directly related to the levels of inflammation before ART is initiated.^[13,14]^

Here, we sought to examine the potential link among HIV persistence, inflammation, and lung damage in PWH on ART. We compared levels of plasma HIV RNA, HIV DNA, and CA RNA in PBMC, determined their association with pulmonary dysfunction and systemic inflammation and examined the contribution of smoking history.

## 2. Methods

### 2.1. Study participants

We enrolled 82 PWH (out of a total of 106 screened) from the Pittsburgh Lung HIV study cohort^[6,15]^ who were 18 to 75 years old and on suppressive ART with plasma HIV RNA levels <50 copies/mL by commercial assay for at least two years. Individuals with other active infections, or significant or uncontrolled systemic diseases were excluded. Demographic and clinical data were collected by standardized participant interview and included age, gender, smoking history, and current ART regimen. Peripheral CD4^+^ T cell count and plasma viral load were confirmed by chart review or direct testing. No participants were excluded unless they were clinically unable to undergo pulmonary function testing.

### 2.2. Pulmonary function testing

Spirometry before and after bronchodilation and diffusing capacity for carbon monoxide (DLco) were done according to established guidelines and procedures.^[16–19]^

### 2.3. Measurement of plasma inflammatory mediators

Plasma cytokines were measured with a Bio-Plex human cytokine, chemokine 17-plex assay kit (Bio-Rad, Hercules, CA): interleukin (IL)-1β, IL-2, IL-4, IL-5, IL-6, IL-7, IL-8, IL-10, IL-12p70, IL-13, IL-17a, Interferon (IFN)-γ, CCL2, CCL4, TNF-α, granulocyte colony-stimulating factor (G-CSF), and granulocyte-macrophage colony-stimulating factor (GM-CSF).

### 2.4. Measures of HIV persistence

HIV nucleic acid from plasma or PBMC was extracted and quantified according to published methods.^[20–24]^ Briefly, samples were lysed with guanidinium-hydrochloride with proteinase-K followed by guanidinium-thiocyanate with glycogen, then precipitated with isopropanol and washed with ethanol. HIV RNA and DNA were quantified by RT-qPCR.

### 2.5. Statistical analysis

Correlation analyses were performed using Pearson or Spearman correlation and adjusted for age and smoking pack-years using partial correlation. Distribution of continuous variables was tested with Shapiro-Wilks test, and the best transformation to normal was used. Analyses were performed in Stata 14.2 (StataCorp, College Station, TX).

### 2.6. Ethics statement

Written informed consent was obtained from each participant. The study protocol was approved by the Institutional Review Boards at the University of Pittsburgh and the University of California San Francisco.

## 3. Results

41% of participants were women, 44% were Caucasian, and the median age was 52 years. Median plasma HIV RNA by single copy assay, which has greater sensitivity than FDA-cleared assays, was 1.4 copies/mL. Median HIV DNA was 298 copies per million PBMC, and median cell-associated HIV RNA was 18 copies per million PBMC. More than half of the cohort had a history of cigarette smoking and approximately half were current smokers. Median CD4^+^ T cell count was 580 cells/mm^3^. Median postbronchodilator forced expiratory volume (FEV1%) predicted was 89%, median postbronchodilator forced vital capacity (FVC%) predicted was 88%, median FEV1/FVC was 81%, and median DLCO% predicted was 79% (Table 1).

**Table 1:** Participant demographics, pulmonary function, and HIV persistence measures.

Participant characteristics (n = 82)	Median (IQR) or n (%)
Age (yr)	52 (46–57)
Female	34 (41)
Race	
Caucasian	36 (44)
African American	41 (50)
Other	5 (6)
Smoking status	
History of smoking	52 (63)
Former smoker	25 (48)
Current smoker	27 (52)
Pack-years smoking (all participants)	7.0 (0–16.3)
Former smoker	10.6 (6.3–29.8)
Current smoker	13.3 (8.8–32.2)
Illicit drug user	28 (34)
CD4+ T cell count (cells/µL)	580 (361–895)
postBD FEV1% predicted	89 (72–103)
postBD FVC% predicted	88 (75–99)
postBD FEV1/FVC - %?	81 (73–85)
DLCO% predicted	79 (68–92)
Plasma HIV-1 RNA (copies/mL)	1.4 (0.7–5.5)
HIV-1 DNA (copies/million PBMC)	298 (134–538)
CA HIV-1 RNA (copies/million PBMC)	18 (0.7–48)

ART = antiretroviral therapy, CA HIV-1 RNA = Cell-Associated HIV-1 RNA, DLCO% predicted = percent single breath diffusing capacity for carbon monoxide adjusted for hemoglobin and carboxyhemoglobin, IQR = interquartile range, PBMCs = peripheral blood mononuclear cells, Post FEV1% predicted = percent predicted postbronchodilator (BD) forced expiratory volume in 1 second, Post FVC% predicted = percent predicted postBD forced vital capacity.

HIV DNA and CA-RNA were positively correlated with the duration and intensity of cigarette smoking (pack-years) (*R* = 0.3, *P* < 0.05 and *R* = 0.4, *P* < 0.01, respectively), and higher HIV DNA in PBMC was associated with being a current smoker (*R* = 0.3, *P* < 0.05) (Table 2). These associations were maintained or strengthened after adjustment for participant age. Plasma HIV RNA was not associated with smoking pack-years. HIV DNA, CA-RNA, and plasma HIV RNA were not significantly associated with any measure of pulmonary function. Age, gender, race, and CD4^+^ T cell count were not associated with any HIV reservoir measurement.

**Table 2:** Correlations of measures of HIV persistence in blood with participant age, smoking, pulmonary function, and peripheral inflammatory mediators.

Pearson correlation, *R*	Plasma SCA * (avg cp/mL) n = 79–80	HIV-1 DNA † (cp/1M PBMC) n = 49–52	CA HIV-1 RNA † (cp/1M PBMC) n = 49–52
Age	–0.04	0.27	–0.01
Current smoker	0.07	**0.30** ‡	0.20
Pack-years †	0.00	**0.30** ‡	**0.40** §
postBD FEV1% predicted	0.01	0.03	0.18
postBD FVC% predicted	0.00	0.06	0.19
postBD FEV1/FVC	0.05	–0.09	0.04
DLCO% predicted	0.03	–0.056	0.16
IL1-β†	0.15	–0.18	–0.27
IL-2†	0.06	–0.04	–0.16
IL-4†	0.15	–0.08	**–0.30** ‡ ∥
IL-5†	-0.09	–0.12	–0.22
IL-6†	0.15	–0.17	–0.27
IL-7*	0.01	–0.04	–0.22
IL-8*	0.15	0.00	–0.13
IL-10†	–0.09	–0.18	**–0.35** ‡ ∥
IL-13†	–0.04	–0.11	**–0.29** ‡ ∥
IL-17†	–0.05	–0.12	–0.25
G-CSF†	–0.05	–0.24	**–0.44** § ∥
GM-CSF†	–0.14	–0.17	**–0.32** ‡ ∥
IL-12*	0.05	–0.11	–0.23
INF-γ*	0.11	–0.08	–0.23
TNF-α†	0.18	0.11	0.06
CCL4*	0.14	–0.04	–0.01

CA HIV-1 RNA = Cell-Associated HIV-1 RNA, DLCO% = percent single breath diffusing capacity for carbon monoxide, G-CSF = Granulocyte colony-stimulating factor, GM-CSF = Granulocyte-macrophage colony-stimulating factor, PBMC = peripheral blood mononuclear cells, Plasma SCA = Single Copy Assay Quantification of Plasma HIV-1 RNA, Post FEV1% = percent predicted postbronchodilator (BD) forced expiratory volume in 1 second, Post FVC% = percent predicted postBD forced vital capacity.

* Natural log.

† Square root.

‡ *P* < 0.05.

§ *P* < 0.01.

∥ The correlations remain significant after adjusting for pack-years.

CA-RNA was negatively correlated with the antiinflammatory peripheral cytokines IL-4 (*R* = –0.3, *P* < 0.05), IL-10 (*R* = –0.35, *P* < 0.05), IL-13 (*R* = –0.29, *P* < 0.05), G-CSF (*R* = –0.44, *P* < 0.01), and GM-CSF (*R* = –0.32, *P* < 0.05), even after adjusting for age and smoking pack-years. Plasma HIV RNA by SCA and HIV DNA were not associated with any peripheral inflammatory mediator, and no measurement of HIV persistence was associated with pro-inflammatory cytokines in plasma^[14]^ (Table 2).

## 4. Discussion

In this cross-sectional study of PWH on long-term ART, we examined the association of ultrasensitive measures of HIV persistence with age, pulmonary function, smoking history, and levels of peripheral inflammatory mediators. Over 50% of PWH are current smokers,^[25]^ so it is essential to understand the impact of smoking on HIV dynamics. To our knowledge, this is the first study to show a direct positive correlation of the duration and intensity of cigarette smoking (current smoking and pack-years) with the level of HIV DNA and CA-RNA in ART-suppressed participants. We observed significant negative correlations between plasma antiinflammatory cytokine levels (IL-4, IL-10, IL-13, G-CSF, GM-CSF) and CA-RNA, but did not observe any correlation between pro-inflammatory mediators and measures of HIV persistence. The disconnect between cellular measures of HIV persistence and plasma HIV RNA with smoking pack-years could be due to activation and clonal expansion of infected cells carrying defective proviruses that are incapable of producing functional virions.^[26,27]^

Several previous studies have shown that HIV is an independent risk factor for pulmonary disease including COPD.^[28–32]^ HIV infection is associated with a lymphocytic alveolitis,^[10]^ and HIV gp120 may directly cause airway damage,^[11]^ suggesting that HIV may independently stimulate pulmonary inflammation. Drummond *et al* found that high plasma HIV RNA levels (>200,000 copies/mL) were associated with obstructive lung disease,^[12]^ and therefore ART could be beneficial to HIV-associated pulmonary disease.^[12,30,33]^ We found no association of any measure of HIV persistence with impaired lung function by FEV1% predicted, FVC% predicted, FEV1/FVC, or DLCO% predicted. The levels of HIV DNA, CA-RNA, and plasma HIV RNA do not seem to be one of the factors that directly influence lung function in PWH on ART despite the relationship with smoking and inflammation.

This study has several limitations. Inflammatory mediators and HIV-1 levels were only assessed in the periphery and not in respiratory samples (e.g., bronchoalveolar lavage), which could lead to different associations with lung dysfunction. The sample size is limited especially when considering the variation involved with pulmonary function testing. It is possible that associations with HIV-1 reservoir measurements could be due to poor ART adherence in the past or other behavioral factors, though inclusion in the study was limited to individuals suppressed on ART for a minimum of two years. Finally, this is a cross-sectional cohort study while longitudinal investigation may provide a better ability to understand the processes leading to lung dysfunction.

These findings support the understanding that pulmonary function can be reduced in PWH^[34]^ as a result of smoking and systemic inflammation,^[6,15,35,36]^ but that HIV persistence does not directly contribute to lung dysfunction or inflammation.^[14]^ HIV infection in the lung has been difficult to examine outside of the macaque model, where HIV-related pulmonary hypertension has been associated with HIV Nef protein in lung mononuclear cells.^[37]^ Although we did not directly measure HIV persistence in lung tissue or bronchoalveolar lavage, there were no associations with circulating markers of HIV persistence, which reduces but does not eliminate the possibility that infection of resident lung cells contributes to lung dysfunction. Expanded longitudinal investigation will be needed to fully understand the processes leading to lung dysfunction in PWH.

## Acknowledgments

A subset of subjects in this cohort was recruited from the Multicenter AIDS Cohort Study (MACS) and the Women’s Interagency HIV Study (WIHS), now the MACS/WIHS Combined Cohort Study (MWCCS). The contents of this publication are solely the responsibility of the authors and do not represent the official views of the National Institutes of Health (NIH). MWCCS (Principal Investigators): Data Analysis and Coordination Center (Gypsyamber D’Souza, Stephen Gange and Elizabeth Golub), U01-HL146240; Northern California CRS (Bradley Aouizerat, Jennifer Price, and Phyllis Tien), U01-HL146242; Pittsburgh CRS (Jeremy Martinson and Charles Rinaldo), U01-HL146208. The MWCCS is funded primarily by the National Heart, Lung, and Blood Institute (NHLBI), with additional co-funding from the Eunice Kennedy Shriver National Institute of Child Health & Human Development (NICHD), National Institute on Aging (NIA), National Institute of Dental & Craniofacial Research (NIDCR), National Institute of Allergy And Infectious Diseases (NIAID), National Institute Of Neurological Disorders And Stroke (NINDS), National Institute of Mental Health (NIMH), National Institute on Drug Abuse (NIDA), National Institute of Nursing Research (NINR), National Cancer Institute (NCI), National Institute on Alcohol Abuse and Alcoholism (NIAAA), National Institute on Deafness and other Communication Disorders (NIDCD), National Institute of Diabetes and Digestive and Kidney Diseases (NIDDK), National Institute on Minority Health and Health Disparities (NIMHD), and in coordination and alignment with the research priorities of the National Institutes of Health, Office of AIDS Research (OAR). MWCCS data collection is also supported by UL1-TR000004 (UCSF CTSA).

## Author contributions

Manuscript preparation: JC, SQ, MN, LH, CR, LK, PT, JM, AM.

Research Assays: JC, SQ, BS.

Clinical Work: MF, CK, LH, PT, AM.

Project Leadership: MN, LH, CR, LK, PT, JM, AM.

Statistical Analyses: RD, MN.
